# Novel Zn metal–organic framework with the thiazole sites for fast and efficient removal of heavy metal ions from water

**DOI:** 10.1038/s41598-023-38523-w

**Published:** 2023-07-15

**Authors:** Akram Karbalaee Hosseini, Azadeh Tadjarodi

**Affiliations:** Research Laboratory of Inorganic Materials Synthesis, Department of Chemistry, Iran University of Science and Technology (IUST), Tehran, 16846-13114 Iran

**Keywords:** Chemistry, Coordination chemistry

## Abstract

Pollution of water by heavy metal ions such as Pb^2+^ and Hg^2+^ is considered as an important issue, because of the potential toxic effects these ions impose on environmental ecosystems and human health. A new Zn-based metal–organic framework, [Zn_2_(DPTTZ) (OBA)_2_] (**IUST-2**), was synthesized through a solvothermal method by the reaction of 2, 5-di (4- pyridyl) thiazolo [5, 4-d] thiazole ligand (DPTTZ), the “V-shape” 4,4'-oxybis (benzoic acid) ligand (OBA) and zinc nitrate (Zn(NO_3_)_2_·6H_2_O). This novel MOF has been characterized by several analysis techniques such as fourier transform infrared spectroscopy (FT-IR), elemental analysis (EA), powder x-ray diffraction (PXRD), thermogravimetry analysis (TGA), differential thermal analysis (DTA), field emission scanning electron microscopy (FE-SEM), Brunauer–Emmett–Teller (BET) surface area analysis and single-crystal X-ray diffraction (SXRD). This 3D MOF was tested for removing Pb^2+^ and Hg^2+^ ions from water. The factors that were investigated on the elimination of Pb^2+^ and Hg^2+^ ions were of pH, adsorption time, and the effect of initial ions concentration. According to the results, this particular Zn-MOF had significant performance in eliminating Pb^2+^ and Hg^2+^ ions from water with a removal efficiency of more than 97% and 87% within 3 min, respectively.

## Introduction

One of the most common sources of water contamination is through industrial sewages and this has been mentioned as a major concern in environmental issues^1^. There are different types of water contaminants such as inorganic and organic pollutants including toxic metals, different types of nutrients, insecticides, chemical fertilizers, detergents, hospital wastewater and persistent organic pollutants (POPs)^2^. Heavy metal ions (Hg^2+^, Pb^2+^, Cd^2+^, Cu^2+^, Cr^3+^, etc.) in water, have been mentioned as a serious threat to human health and living organisms because of their dangerous effects on different body organs^3, 4^. Specifically, mercury ion (Hg^2+^) and lead ion (Pb^2+^) contaminations in water resources, can lead to various disorders including central nervous system disorder, kidneys dysfunction, and stomach problems^5, 6^. Thus, the importance of detection and removal of these toxic heavy metal ions from water has been greatly studied in order to have a healthier life and environment^7, 8^.

Recently, the emergence of metal–organic frameworks (MOFs) and coordination polymers (CPs) porous materials has been greatly regarded as a mean to tackle this problem. These porous structures which are basically constructed of metal ions or metal clusters and organic ligands, have gotten remarkable range of applications as adsorbents^9–13^. MOFs have various properties which make them a good candidate in adsorption including large surface areas, ultrahigh porosity, distributed functional sites, and high thermal/chemical stability^14, 15^. Specific functional groups with sulfur and nitrogen atoms on organic linkers of MOFs through Hg–S interactions or Hg–N interactions have created suitable materials as sorbents for Pb^2+^ and Hg^2+^ions^16–18^. Among them, Thiazole, a five-membered heterocyclic compound, having N and S atoms can adsorb heavy metal ions based on coordination interaction^19, 20^. Organic ligands containing the thiazole ring have been less used in the synthesis of MOFs and CPs.

In this research, 2, 5-di (4- pyridyl) thiazolo [5, 4-d] thiazole (DPTTZ) ligand having thiazole ring was used as linker to synthesize a novel Zn-MOF, [Zn_2_ (DPTTZ) (OBA)_2_] (**IUST-2**) [OBA = 4,4'-oxybis (benzoic acid)], later to be tested for the removal of heavy metal ions from water. In this study, the influence of parameters such as contact time, pH parameters, and also the concentrations of lead and mercury ions in the solutions, were evaluated. This work shows that MOFs containing thiazole ring can play a very effective role in the removal of heavy metal ions from water.

## Experimental

### Materials and apparatus

All chemicals used in this research were of analytical laboratory grade, provided by well-known commercial sources, and used as received. 2, 5 -di (4- pyridyl) thiazolo [5, 4-d] thiazole (DPTTZ) was synthesized by reported procedure^21^. Powder X-ray diffraction (PXRD) patterns were recorded on Philips X’pert diffractometer. Elemental analysis was performed with a CHNS Thermo Finnigan Flash 1112 series elemental analyzer. The absorption spectroscopy FT–IR was measured in the 400–4000 cm^−1^ range, by the KBr disc technique on a Shimaduz FT-IR-8400 spectrometer. Thermal gravimetric analysis (TGA) and differential thermal analysis (DTA) were carried out with Perkin Elmer Pyris 1 thermo gravimeter at 10 °C.min^−1^ heating rate under the argon (Ar) atmosphere. Field emission scanning electron microscopy (FE-SEM) images were taken on the FE-SEM TESCAN MIRA3 microscope. Atomic absorption spectrometry (AAS) on a Shimadzu 6300 AA instrument was used to detect the metal ion concentration in aqueous solutions. Nitrogen adsorption–desorption measurements (BET method) were performed at liquid nitrogen temperature (− 196 °C) with a Micromeritics ASAP 2020 adsorption instrument.

### Single crystal X-ray diffraction

Crystallographic data for the **IUST-2** were collected using Mo Kα radiation (*λ* = 0.71073 Å) on a Marresearch 345 dtb diffractometer equipped with an image plate detector. The programs used to solve and refine the structure were SHELXT 2018/2 (Sheldrick, 2018) and SHELXL2016/6 (Sheldrick, 2016), respectively^22^. Data reduction and cell refinement were carried out with the Automar software package (3.3a, 2015). Non-hydrogen atoms were refined anisotropically. Hydrogen atoms were added at ideal positions and refined using a riding model. Table S1 summarized the single-crystal x-ray diffraction data and structure refinement for **IUST-2**.

### Preparation of [Zn_2_ (DPTTZ) (OBA)_2_] (IUST-2)

A mixture of Zn(NO_3_)_2_^.^6H_2_O (0.010 g, 0.034 mmol), 4,4'-oxybis (benzoic acid) (0.009 g, 0.034 mmol), 2, 5-di (4- pyridyl) thiazolo [5, 4-d] thiazole (0.005 g, 0.017 mmol) and N,N-dimethylformamide (7 mL) was sealed in a 15 mL stainless steel reactor with Teflon liner and directly heated to 120 °C for 72 h and then cooled to 25 °C during 12 h. The single clear yellow crystals suitable for X-ray diffraction experiments were obtained with a good yield of 71.8%. FT-IR (KBr, cm^-1^): 3424(m), 3057(m), 2923(m), 1676(s), 1635(s), 1610(s), 1593(s), 1499(s), 1396(s), 1230(s), 1158(s), 1091(m), 1065(s), 1012(s), 877(s), 830(s), 782(s), 696(s), 661(s), 618(s), 504(s), 444(s). Elemental Anal. calc. for C_42_H_24_N_4_O_10_S_2_Zn_2_: C, 53.69; H, 2.57; N, 5.96; S, 6.83%. Found: C, 53.46; H, 3.34; N, 6.88; S, 6.62%.

### Adsorption experiments

A series of batch absorption experiments were performed to determine the capability of the **IUST-2** to adsorb targeted heavy metal ions (i.e. Pb^2+^ and Hg^2+^) from aqueous solutions. To do this, 5 mg of **IUST-2** was placed into 25 mL of aqueous solutions with varied concentrations of targeted heavy metals and stirred at room temperature. The pH and adsorption time were investigated on the elimination of Pb^2+^ and Hg^2+^ cations onto the **IUST-2**. HCl/NaOH solution (0.1 M) was used to regulate the solution pH. The remaining concentration of these metal ions in each of the stock solutions were determined by atomic absorption spectrometry.

To investigate the kinetics of the sorption process, 5 mg **IUST-2** was placed in a round-bottomed flask and then left to interact with 25 mL of aqueous solutions at 200 mg L^−1^ concentration of Pb^2+^ and Hg^2+^, separately, for various time intervals between 0.5 to 120 min. Moreover, the maximum adsorption capacities of **IUST-2** for lead and mercury ions were estimated separately, by adsorption isotherms in a series of Pb^2+^ and Hg^2+^ solutions with the concentration range of 50–600 mg L^−1^. In all the aforementioned tests, pH values were set to be 5 and 4 for lead and mercury solutions, respectively.

## Results and discussion

### Crystal structure and characterization of IUST-2

**Figure 1:**
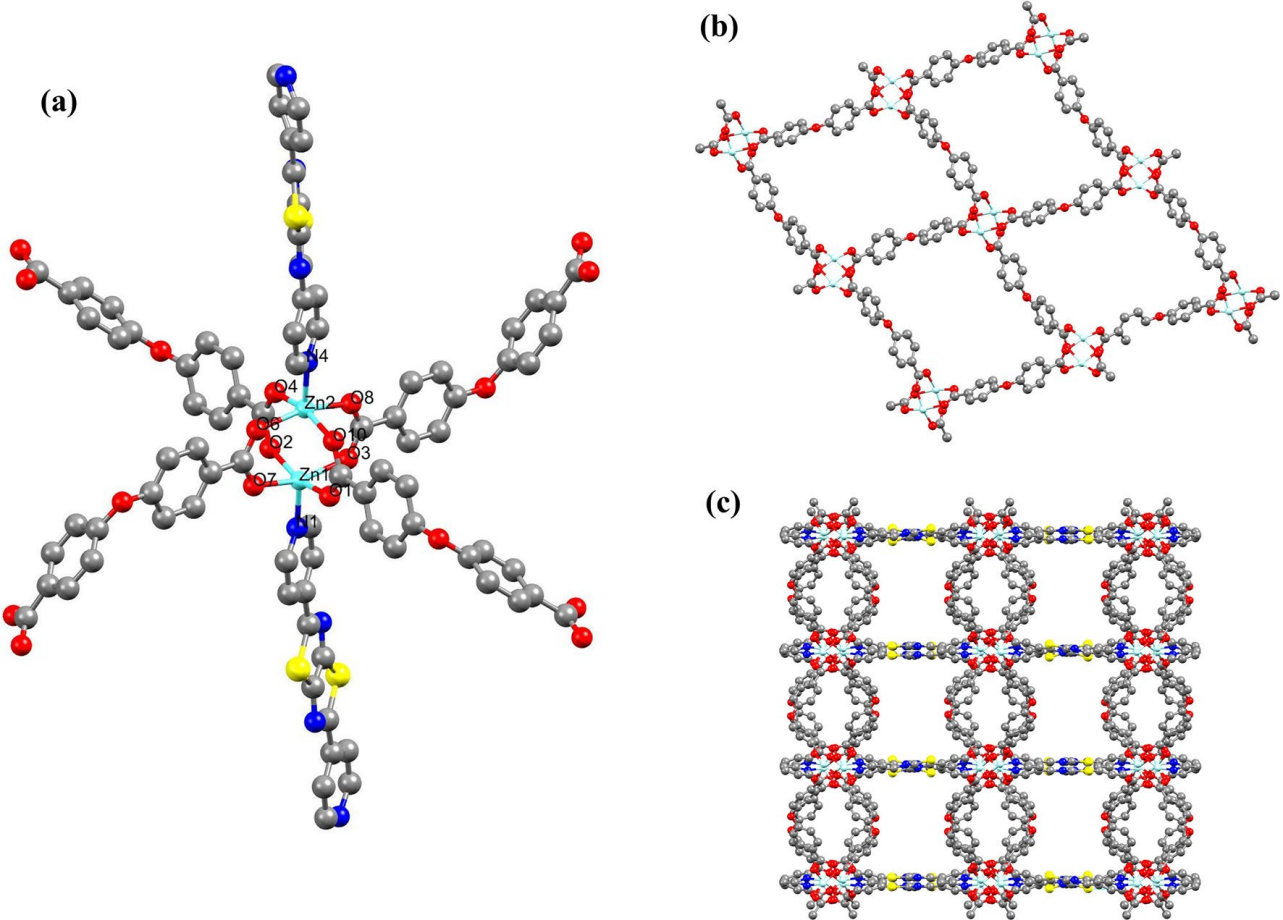
(**a**) The Zn^2+^ ion Coordination environment in **IUST-2**; Symmetry codes: (i) − *x* + 1/2, − *y* + 3/2, *z* + 1/2; (ii) − *x* + 1/2, − *y* + 1/2, *z* − 1/2; (iii) − *x* + 1, *y*, − *z* + 1/2; (iv) − *x*, − *y* + 1, − *z* + 2; (v) − *x* + 1/2, − *y* + 3/2, *z* − 1/2; (vi) − *x* + 1/2, − *y* + 1/2, *z* + 1/2, (**b**) 2D sheet of {Zn_2_(OBA)_2_}_*n*_ along the *bc* plane, (**c**) View of 3D framework of the **IUST-2** along c axis.

According to the single crystal X-ray analysis, **IUST-2** crystallizes in an orthorhombic crystal system and the space group is *P*bcn with the asymmetric unit comprising of two Zn^2+^ ions, one DPTTZ ligand and two OBA ligands. As illustrated in Fig. 1a, each Zn^2+^ ion displays a square-pyramidal coordination geometry surrounded by four carboxylate O atoms from four OBA ligands at the equatorial positions and one pyridine N atom from one DPTTZ ligand at the axial position. The Zn − O bond lengths are in the range from 2.254(1) to 2.361(2) Å and the Zn − N bond distance is 2.271(2) Å. The O − Zn − O and N − Zn − O angles are in the range of 86.36(18)° − 161.79(15)° and 94.48(17)° − 103.81(17)°, respectively. From a crystallography point of view, four carboxyl groups from four OBA ligands, act as a bridging ligands to link independent Zn^2+^ ions, making a paddle-wheel shaped secondary building unit (SBU) Zn_2_(CO_2_)_4_. The carboxylate groups of OBA ligands adopting bismonodentate coordination modes μ_2_–η^1^:η^1^ link Zn nodes to form a two- dimensional wave-like sheets expanded by DPTTZ ligands, eventually resulting a 3D structure (Fig. 1b and c).

### PXRD

The phase purity of **IUST-2** was checked by powder X-ray diffraction (PXRD) experiments. The simulated and experimental PXRD patterns of the **IUST-2** correspond to each other, demonstrating the phase purity of the **IUST-2** crystals prepared using the solvothermal method. Comparison of powder x-ray diffraction patterns before and after the activation process with methanol showed that the **IUST-2** structure remains intact after activation (Fig. S1). Considering the target application for **IUST-2** as an adsorbent to remove metal ions in aqueous media, the water-stability of the IUST- 2 was further studied. The **IUST-2** was immersed in water at RT for 48 h. Slight changes in the PXRD pattern were observed after 48 h of soaking in water, which returned to the initial state, after 24 h soaking in DMF (Fig. S2). This observation is possibly attributed to the MOFs’ breathing effect^23^.

### TGA and DTA

In order to investigate the thermal stability of **IUST-2**, thermogravimetric and differential thermal analysis experiments were carried out under the Ar atmosphere (Fig. S3). A weight loss with a mild rate (approximately 20%) in the beginning, corresponds to the evaporation of trapped solvent molecules inside the **IUST-2** during the washing process. Decomposition and pyrolysis of the **IUST-2** starts with an endothermic process at 363 °C, which was the main thermal loss of 72%. The remaining weight (approximately 8%) may be attributed to the formation of zinc oxide, or zinc sulfide. This indicates that **IUST-2** has rather good thermal stability.

### BET

To measure N_2_ adsorption/desorption isotherm, a sample **IUST-2** was degassed at 110 °C for 4 h. Fig. S4 shows that the N_2_ adsorption–desorption isotherm of the **IUST-2** is of type IV, according to the IUPAC classification, having an H3 type hysteresis loop. The Brunauer–Emmett–Teller (BET) specific surface area of the **IUST-2** is 105.636 m^2.^g^−1^. The pore volume and average pore size are 0.081 cm^3.^g^−1^ and 30.553 Å, respectively.

### FE-SEM

Figure S5 shows the FE-SEM images of **IUST-2** obtained by the solvothermal process. As could be observed from these figures, **IUST-2** has a nanostructure with an average diameter of 31.4 nm.

### Selective adsorption behaviors

**Figure 2:**
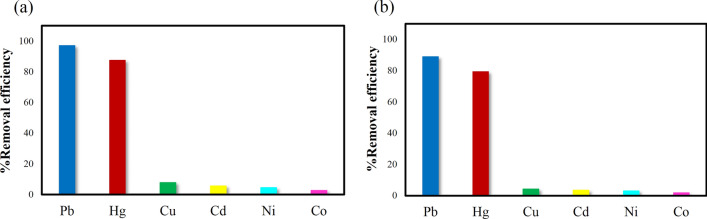
(**a**) The removal efficiency of heavy metal ions for the **IUST-2**: (**a**) on single solutions, (**b**) on coexisting solutions.

Batch sorption tests on an aqueous solution with a range of different heavy metal ions such as (Hg^2+^, Pb^2+^, Cu^2+^, Cd^2+^, Ni^2+^, Co^2+^) were carried out to determine the capacity and behavior of **IUST-2** in selective adsorption. From the results, it can be noticed that **IUST-2** shows much higher removal efficiency in the presence of the Pb^2+^ (%97.08) and Hg^2+^ (%87.55) compared to other heavy metal ions, when applied to single solutions of different ions (Fig. 2a). In another test, and to investigate the interfering effects, the removal efficiency was studied by the **IUST-2** on a mixed aqueous solution of different heavy metal ions, with a concentration of 100 mg^.^L^−1^ each. Still, the removal efficiency was much higher for Pb^2+^ and Hg^2+^ (88% and 79%, respectively), even in the presence of other interfering ions in the solution (Fig. 2b). It can be observed that **IUST-2** revealed excellent uptake performance toward lead and mercury ions, which is assumed to be in relevance to thiazole ring. Thiazole is a unique heterocyclic compound containing sulfur and nitrogen atoms. Because of the strong affinity between N and S atoms of the thiazole ring with lead and mercury ions, compared to other background metal ions, selective removal for Pb^2+^ and Hg^2+^ is outperformed^24–28^.

### The pH effect

**Figure 3:**
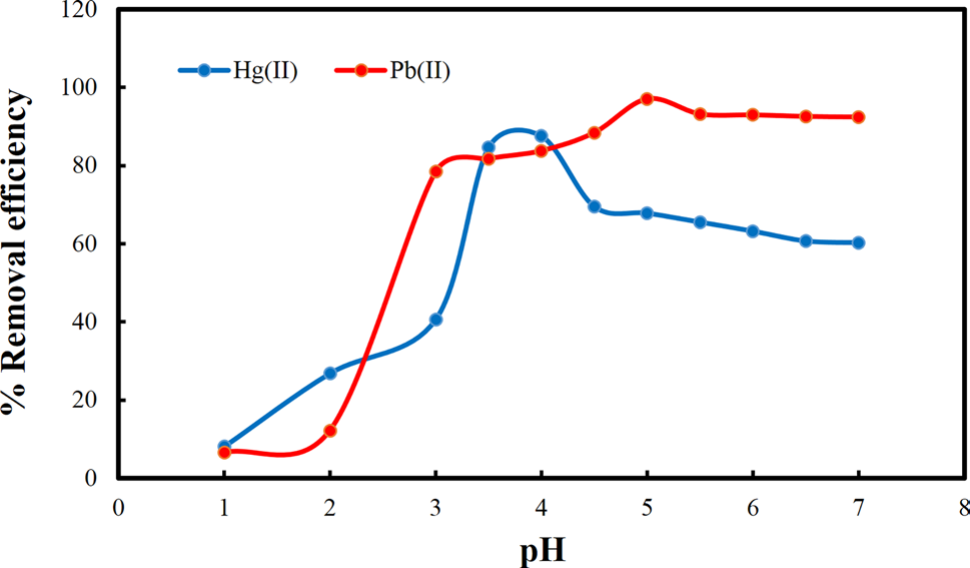
Influence of pH on the removal of lead and mercury ions using the IUST-2.

The pH is a key variable that can both controls and affect the adsorption process. The pH of the solution may have effects on the surface charge of the adsorbate as well as on the adsorbent. Therefore, the impact of the pH ranging from 1.0 to 7.0 on the adsorption of Pb^2+^ and Hg^2+^ ions was investigated (Fig. 3). At pH < 3.5, the ability of the **IUST-2** to remove heavy metal ions was degraded, because hydrogen ions can compete and coordinate better on the available adsorption sites on **IUST-2**. The ability of the **IUST-2** to remove lead and mercury ions is the highest at pH = 5 and 4, respectively. With the pH value increasing, the concentration of hydrogen ions is diminished, and stronger electrostatic interaction occurs between metal ions (Pb^2+^ and Hg^2+^) and active sorption sites of the **IUST-2**. As a result, the optimum pH values of 5.0 and 4.0 were chosen for lead and mercury removal experiments, respectively, throughout this study.

### The effect of concentration

**Figure 4:**
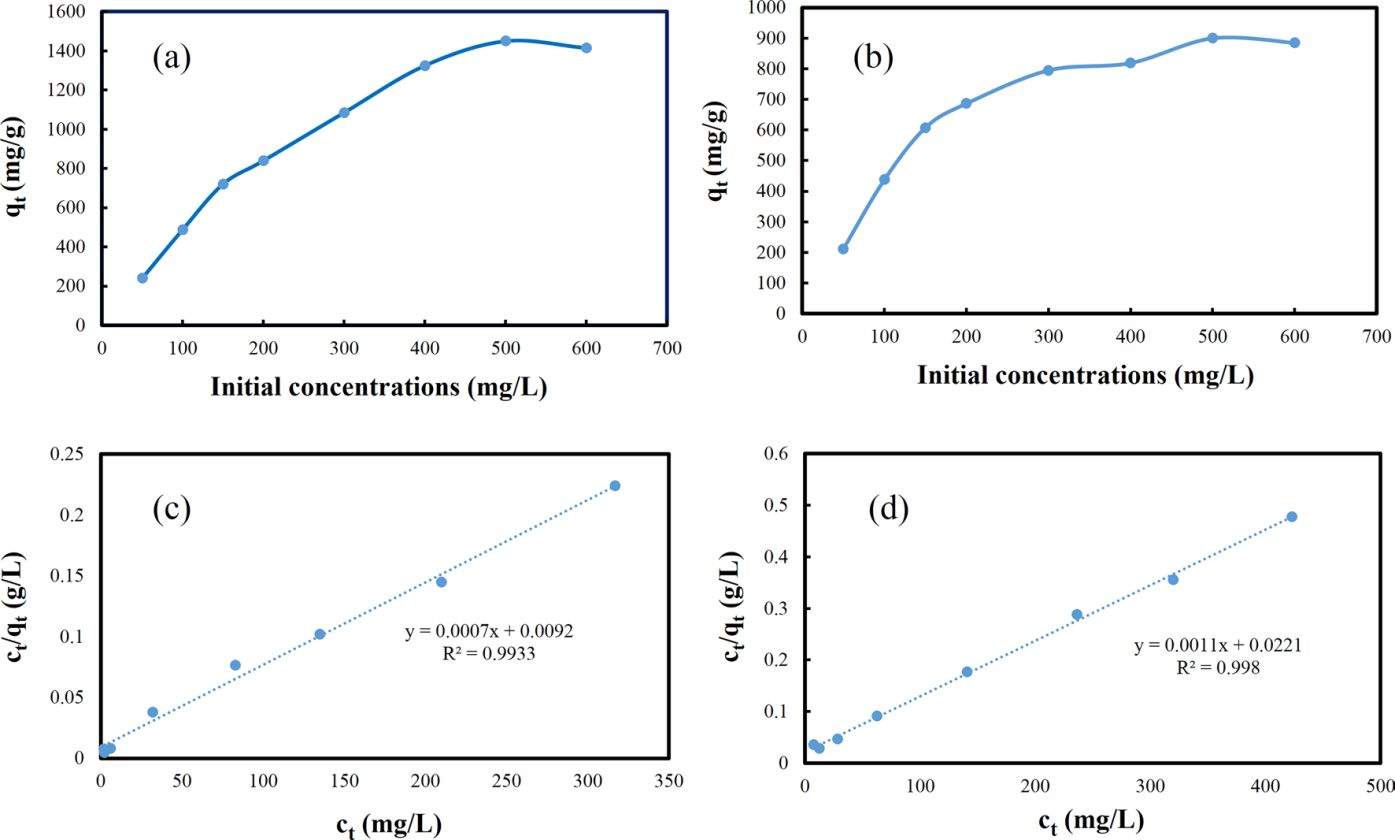
The adsorption isotherm curves for the **IUST-2** at different initial concentrations: (**a**) Pb^2+^, (**b**) Hg^2+^, and fitted lines with the Langmuir isotherm model: (**c**) Pb^2+^, (**d**) Hg^2+^.

The influence of initial Hg^2+^ and Pb^2+^ concentrations on the adsorption efficiency by the **IUST-2** was examined at room temperature. The adsorption isotherm curves are demonstrated for the adsorption of Pb^2+^ (Fig. 4a) and Hg^2+^ ions (Fig. 4b) at different initial concentrations (50–600 mg L^−1^) by 5 mg of the **IUST-2** in 25 mL solution of heavy metal ions. It was observed that the adsorption capacity of the **IUST-2** for Pb^2+^ and Hg^2+^ increased gradually to 1450 mg g^−1^ and 900 mg g^−1^_,_ respectively, at an initial concentration of 500 mg L^−1^. These values for adsorption capacity are so much larger compared to other reported values for other MOFs^29–33^.

The experimental adsorption isotherms data fitted well with the Langmuir model, which is evident from the high correlation coefficient (R^2^ = 0.9933 for Pb^2+^ and 0.9980 for Hg^2+^) values. The equation of the Langmuir isotherm is given as Eq. (1):

1 $$\frac{{c}_{t}}{{q}_{t}} = \frac{1}{({q}_{e}.b)}+ \frac{{c}_{t}}{{q}_{e}},$$

where q_t_ (mg g^−1^) represents the adsorption capacity at time t, c_t_ (mg L^−1^) is the metal ion concentration at time t, b (L mg^−1^) is related to the energy of adsorption that demonstrates the affinity between the solute and the adsorbent, and q_e_ (mg g^−1^) represents the maximum monolayer adsorption capacity.

Since the Langmuir model is compatible to the adsorption behavior, it is concluded that the distribution of active sites on the surface of **IUST-2** is homogeneous^34, 35^. The results of the Langmuir model for lead (Fig. 4c) and mercury (Fig. 4d) ions adsorption on the **IUST-2** is exhibited in Table S2. The theoretical maximum monolayer adsorption (q_e_) of Pb^2+^ and Hg^2+^ ions were computed to be 1430 and 900 mg g^−1^, respectively, which are very close to the values of experimental maximum adsorption capacity (1450 and 900 mg.g^−1^).

### Adsorption kinetics and mechanism

**Figure 5:**
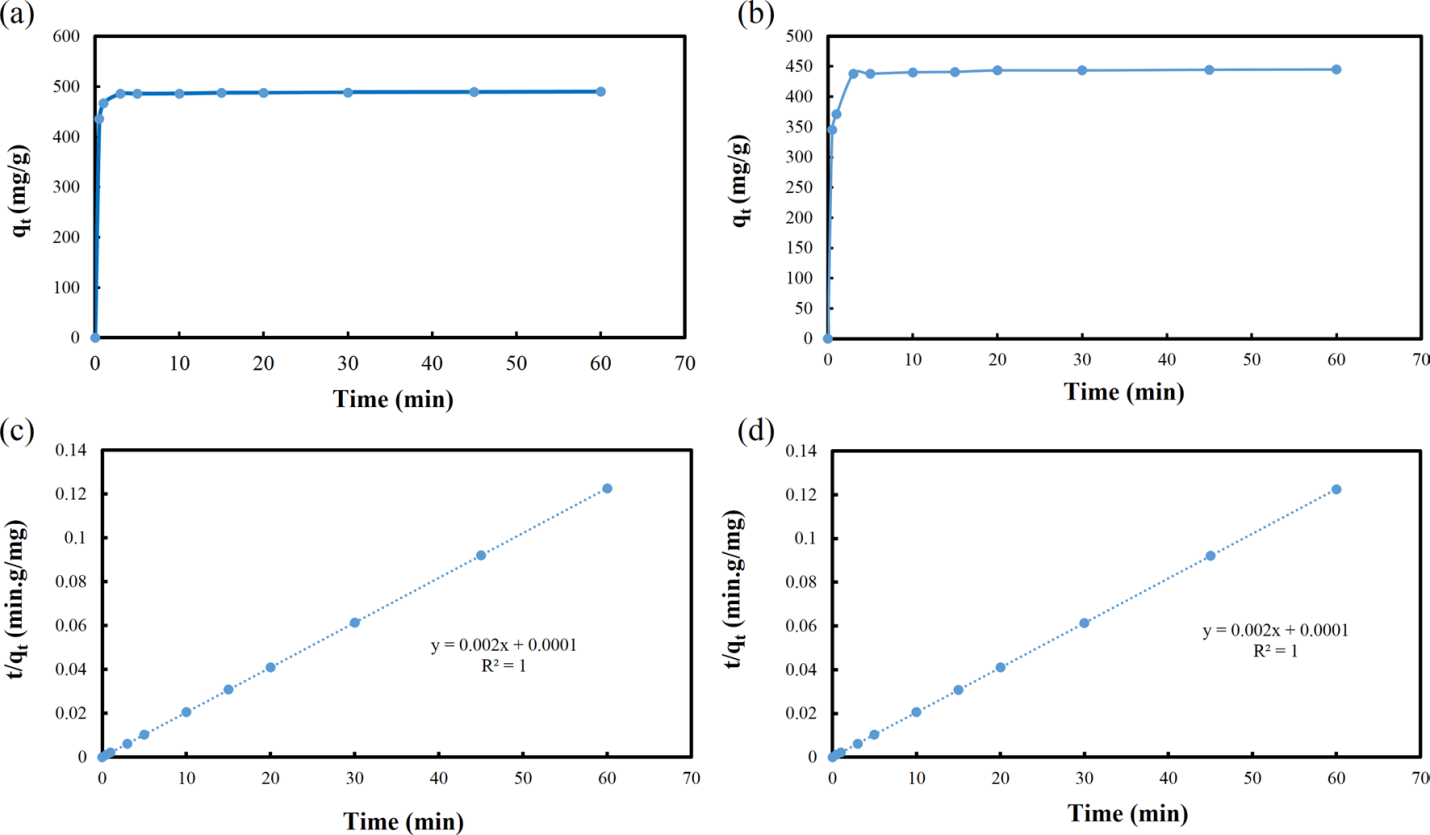
Time-dependent adsorption capacity of **IUST-2** toward: (**a**) Pb^2+^, (**b**) Hg^2+^, and Adsorption kinetics fitted with pseudo-second-order models: (**c**) Pb^2+^, (**d**) Hg^2+^.

As shown in Fig. 5a and b, variation of the removal capacity for Pb^2+^ and Hg^2+^ ions on the **IUST-2** with time were achieved. Due to the presence of specific functional groups on the **IUST-2** and high metal ions affinity for the sulfur and nitrogen of the thiazole ring, the removal of targeted ions is very quick and the maximum adsorption capacity is acquired after 3 min. The activated **IUST-2** (5 mg) was placed in an aqueous solution of Pb(NO_3_)_2_ and HgCl_2_ (25 mL, 100 mg L^−1^ initial concentration, pH = 5 and 4 for lead and mercury ions, respectively) and then the achieved data were fitted with pseudo-second order kinetic model (Fig. 5c for lead and Fig. 5d for mercury ions) using the Eq. (2):

2 $$\frac{t}{{q}_{t}} = \frac{1}{({k}_{2}.{q}_{e}^{2})}+ \frac{t}{{q}_{e}},$$

where q_e_ and q_t_ (mg^.^g^−1^) are the removal capacity at equilibrium and time t (min), respectively; k_2_ (g (mg min)^−1^) is the pseudo-second order rate constant of the adsorption rate. The high coefficient of determination (R^2^˃ 0.9999) value was obtained, suggesting that the pseudo-second-order model was suitable for the adsorption kinetics of the **IUST-2** and sorption process was mostly governed by chemical reactions between the metal ions and the active adsorption sites of the **IUST-2** (Table S3)^36, 37^.

**Figure 6:**
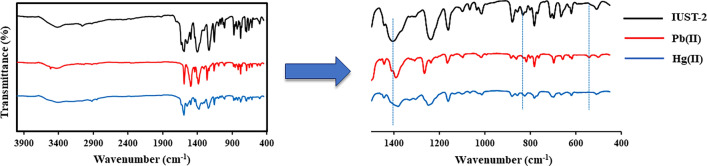
The FT-IR spectra; for the **IUST-2** before and after the adsorption Pb^2+^ and Hg^2+^ ions.

To examine the mechanism of Hg^2+^ and Pb^2+^ ions sorption, the FT-IR spectrum of **IUST-2** before and after the adsorption of metal ions was studied (Fig. 6). There are obvious changes in the infrared spectra of C − S and C − N vibrations of thiazole ring. There are strong interactions between Hg^2+^ and Pb^2+^ ions with S and N atoms of the thiazole ring that could limit C − S and C − N vibrations and consequently decrease their vibrational frequency. The peak at 833 cm^−1^ assigned to C − S stretching vibration that showed a red shift of the 833 cm^−1^ peak to 820 cm^−1^ peak and 824 cm^−1^ peak after treatment with Pb and Hg ions, respectively. Moreover, a significant red shift from 1407 cm^−1^ to 1392 cm^−1^ and 1384 cm^−1^ was observed for the characteristic C − N stretching vibration after the adsorption process, for lead and mercury ions, respectively, which confirmed the coordination of Pb^2+^ and Hg^2+^ ions to the nitrogen of the thiazole ring. Also, a new bond has appeared in 542 cm^−1^, which can attribute to the Pb–O vibration^38^. The Hg-O bond for mercury does not exist in the FT-IR spectrum, so the reason for the increase in the percentage of removal efficiency of **IUST-2** for lead compared to mercury can be attributed to the presence of more adsorption sites for lead. The results of the powder x-ray diffraction before and after the adsorption process reveal that the **IUST-2** can retain its crystallinity and structure after the adsorption of Pb^2+^ and Hg^2+^ metal ions, so the possibility of structural collapse should be removed (Fig. S6).

### Reusability

**Figure 7:**
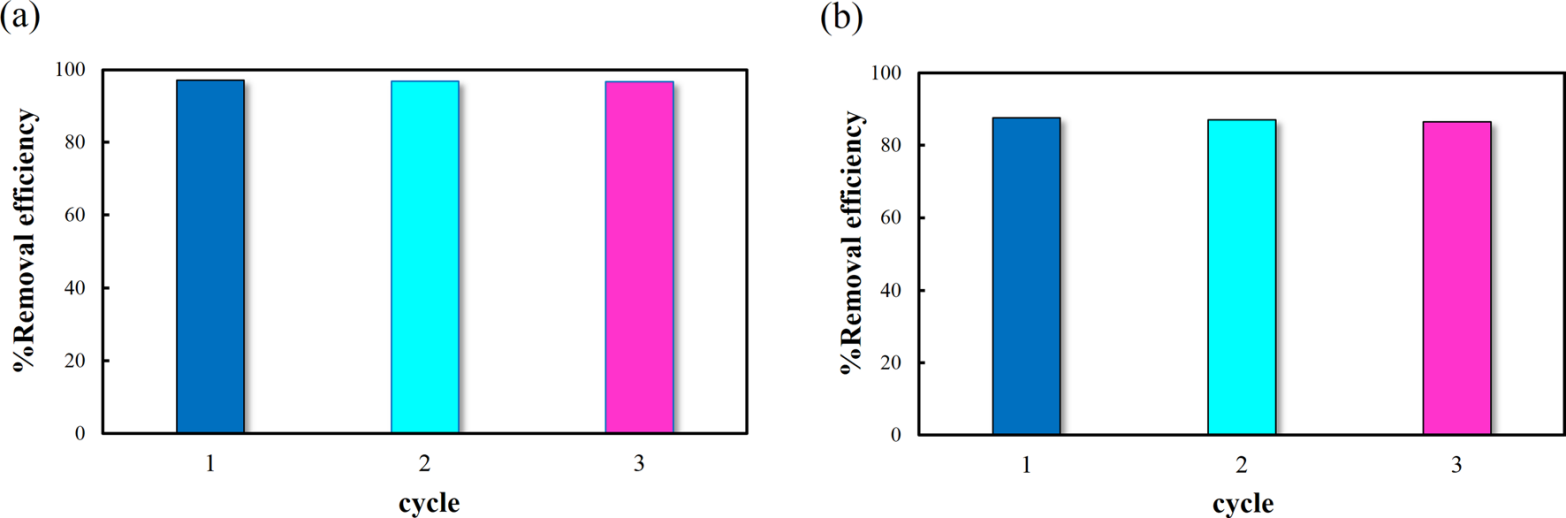
Reusability experiments of **IUST-2** implemented with (**a**) Pb(II) ions, (**b**) Hg(II) ions.

As a general rule, reusability and stability are two essential factors for adsorbent to work effectively. When the adsorption process was finished, the **IUST-2** was regenerated by using an EDTA.2Na solution. Three cycles of adsorption/desorption were carried out to evaluate the reusability of the **IUST-2** and this process was monitored with AAS. Figure 7 demonstrates that the **IUST-2** possesses reversibility in the process of removing Pb^2+^ and Hg^2+^ ions.

### BET of IUST-2 before and after removal of targeted metal ions

The N_2_-BET analysis represents that the surface area of the pristine **IUST-2** is 105.63 m^2^/g m^2^/g. The comparison of the BET surface area of virgin **IUST-2** and **IUST-2** after the adsorption of lead and mercury ions from the water is seen to have decreased from 105.63 m^2^/g to 6.33 m^2^/g, and 18.298 m^2^/g, respectively (Table S4). Also, a decrease was observed in the total pore volume from 0.081 to 0.020 cm^3^/g, and 0.066 cm^3^/g for the **IUST-2** after the adsorption of Pb (II) and Hg (II) ions, respectively (Table S4). This might result from filling the volume of the pores. Hence, it can be concluded that **IUST-2** is a good absorbent towards lead and mercury ions from the aqueous solution. In addition, the results of the BET surface area and pore volume after the removal of metal ions confirmed the reusability of **IUST-2**.

### Comparison with other MOF-based adsorbents

Table 1 indicates the maximum sorption capacity of **IUST-2** for the removal of lead and mercury ions from water compared to other MOF-based adsorbents in the literature. According to Table 1, the maximum sorption capacities of **IUST-2** for Pb (II) and Hg (II) are higher than that of most other MOF-based adsorbents reported in the literature. Totally, these results demonstrated that **IUST-2** is a promising adsorbent for the effective removal of Pb (II) and Hg (II) ions from polluted water.

**Table 1:** The compared results of the sorption capacity of **IUST-2** for Pb (II) and Hg (II) ions with different absorbents based on MOFs.

Absorbent	Adsorption capacities of Pb (II) (mg g^−1^)	Adsorption capacities of Hg (II) (mg g^−1^)	Ref
TMU-31	909	476	^38^
Magnetite nanoparticles@Fe-BTC MOF	147	155	^39^
Fe_3_O_4_-ZrMOF	397	431	^40^
Zn-MOF	1097	32	^26^
[(ZnBTA_0.5_BPP·5H_2_O)n]	132	348	^41^
Cd-CP	450	545	^42^
**IUST-2**	1450	900	This work

## Conclusions

In conclusion, metal–organic frameworks containing the thiazole ring can be a good option for the adsorption of heavy metal ions. In this article, a new Zn-MOF, the **IUST-2**, based on the thiazole ligand was synthesized by solvothermal method. According to the single crystal x-ray diffraction, the **IUST-2** is a 3D bipillared-layer framework structure. The **IUST-2** displayed remarkable application in the adsorption of lead and mercury ions from water. The removal efficiency as high as 97% and 87% was obtained for Pb^2+^ and Hg^2+^ ions at an initial concentration of 100 mg L^−1^ after 3 min, respectively. Maximum adsorption capacity is 1450 mg g^−1^ for Pb^2+^ and 900 mg g^−1^ for Hg^2+^ ions. The results from Langmuir and pseudo-second order rate models reveal the removal of metal ions by the **IUST-2** is a monolayer adsorption by the interaction between the active adsorption sites of the **IUST-2** and Pb^2+^ and Hg^2+^ ions. This study indicates that metal–organic frameworks based on thiazole ligands can be good adsorbents for mercury and lead ions elimination of aqueous solution.

## Supplementary Information

Supplementary Information.

## Data Availability

All data generated or analyzed during this study are included in this published article [and its supplementary information files]. A CCDC Deposition Number 2181756 contain the supplementary crystallographic data. This data can be obtained free of charge from the Cambridge Crystallographic Data Center via the joint CCDC/FIZ Karlsruhe deposition service www.ccdc.cam.ac.uk/structures.
