# Supplementary material for: Novel Zn metal–organic framework with the thiazole sites for fast and efficient removal of heavy metal ions from water
Source: Sci Rep. 2023 Jul 15;13:11430. doi: 10.1038/s41598-023-38523-w (PMC10349873; doi:10.1038/s41598-023-38523-w)
Supplement: Supplementary file 1 — Supplementary Information. [file 41598_2023_38523_MOESM1_ESM.pdf]

Supplementary information

Novel Zn metal-organic framework with the thiazole sites for fast and efficient removal of heavy metal ions from water

Akram Karbalaee Hosseini, Azadeh Tadjarodi

Research Laboratory of Inorganic Materials Synthesis, Department of Chemistry, Iran University of Science and Technology (IUST), 16846-13114 Tehran, Iran

Corresponding author: *Tel: +98(21) 77240517; Fax: +98(21) 77491204.*

Email: *tajarodi@iust.ac.ir (A. Tadjarodi)*

Table of contents

*Fig. S1. PXRD patterns for **IUST-2**: simulated, solvothermal, activated **IUST-2**.*....................1

*Fig. S2. PXRD patterns for **IUST-2**: (a) simulated, (b) solvothermal, (c) immersed sample in water, and (d) immersed sample in DMF after immersing in water.* 1

*Fig. S3. TGA and DTA diagrams of **IUST-2**.* 2

*Fig. S4. Nitrogen adsorption–desorption isotherms for **IUST-2**.*...2

*Fig. S5. FE-SEM images for **IUST-2**.*...3

*Fig. S6. The PXRD patterns; for **IUST-2** before and after Pb(II) and Hg(II) adsorption* 3

*Fig. S7 Nitrogen adsorption–desorption isotherms for **IUST-2**: (a) after the adsorption of Pb (II), (b) after removal Pb (II)* 4

*Fig. S8 Nitrogen adsorption–desorption isotherms for **IUST-2**: (a) after the adsorption of Hg (II), (b) after removal Hg (II) adsorption*...4

*Table S1. Data Collection and Refinement Parameters for Single Crystal Analysis for **IUST-2**.* 5

*Table S2. Fitting results of Langmuir model for **IUST-2**.* 6

*Table S3. Fitting results of pseudo-second-order model for **IUST-2**.* 6

*Table S4 BET of **IUST-2** before and after removal of targeted metal ions*................................6

*Table S5. Selected bond lengths (Å) of the **IUST-2**.* 6

*Table S6. Selected bond angles (^{0}) of the **IUST-2**.* 7

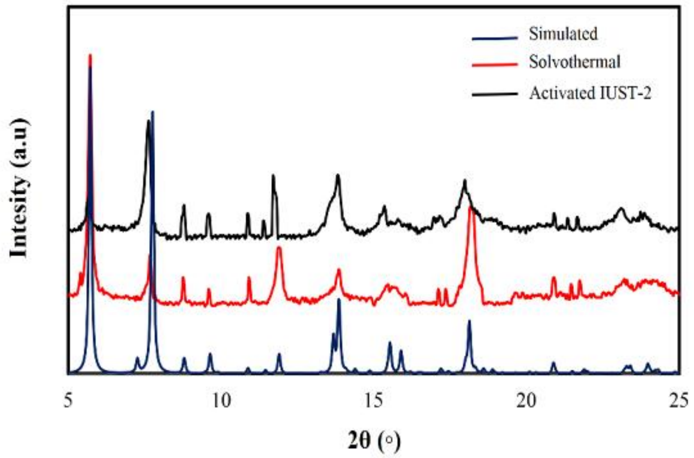

Fig. S1 *PXRD patterns for* ***IUST-2****: simulated, solvothermal, activated* ***IUST-2***

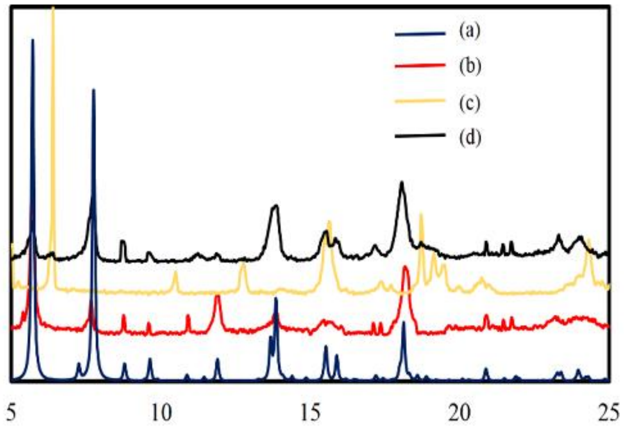

Fig. S2 *PXRD patterns for* ***IUST-2****: (a) simulated, (b) solvothermal, (c) immersed sample in water, and (d) immersed sample in DMF after immersing in water*

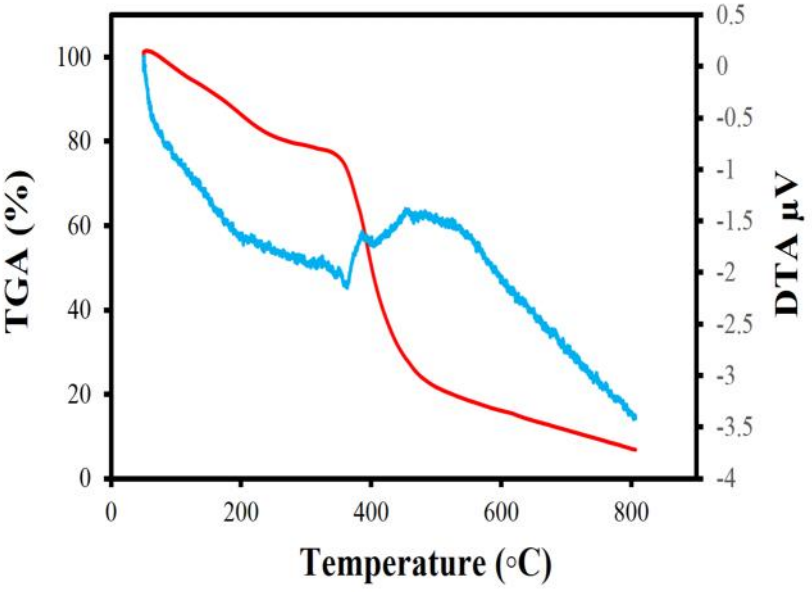

Fig. S3 *TGA and DTA diagrams of* ***IUST-2***

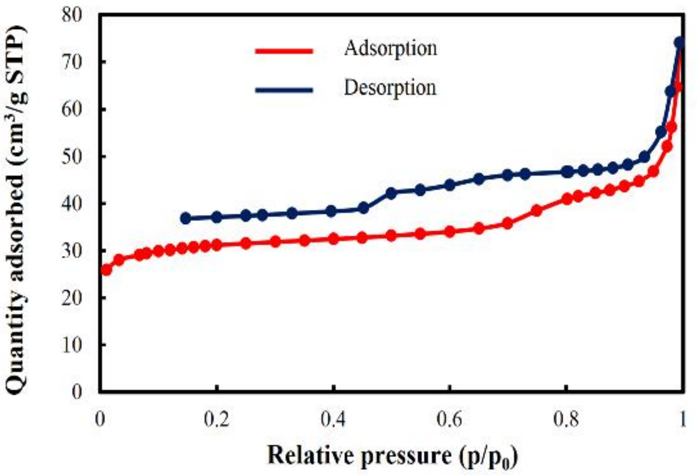

Fig. S4 *Nitrogen adsorption–desorption isotherms for* ***IUST-2***

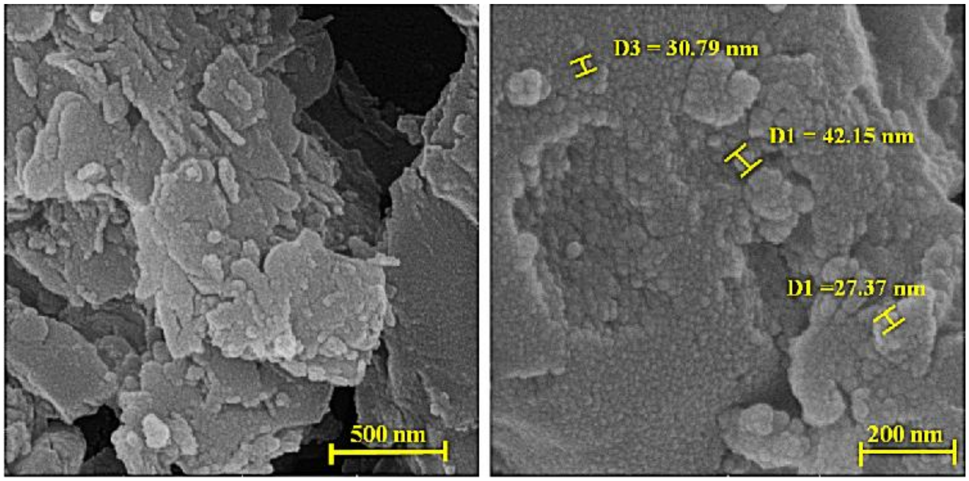

Fig. S5 *FE-SEM images for* ***IUST-2***

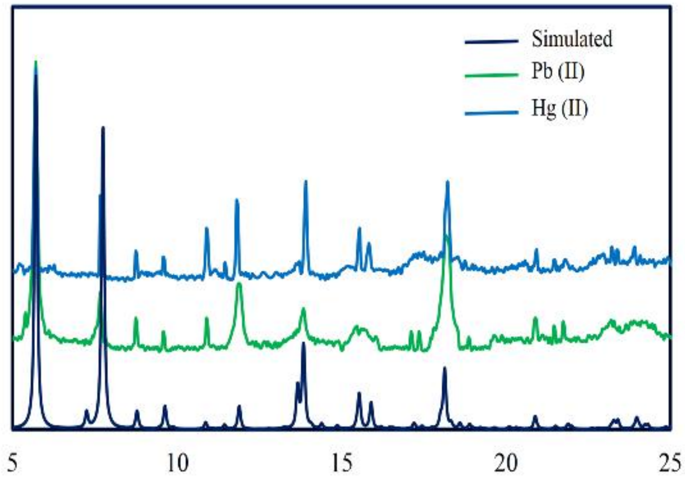

Fig. S6 *The PXRD patterns; for* ***IUST-2*** *before and after Pb(II) and Hg(II) adsorption*

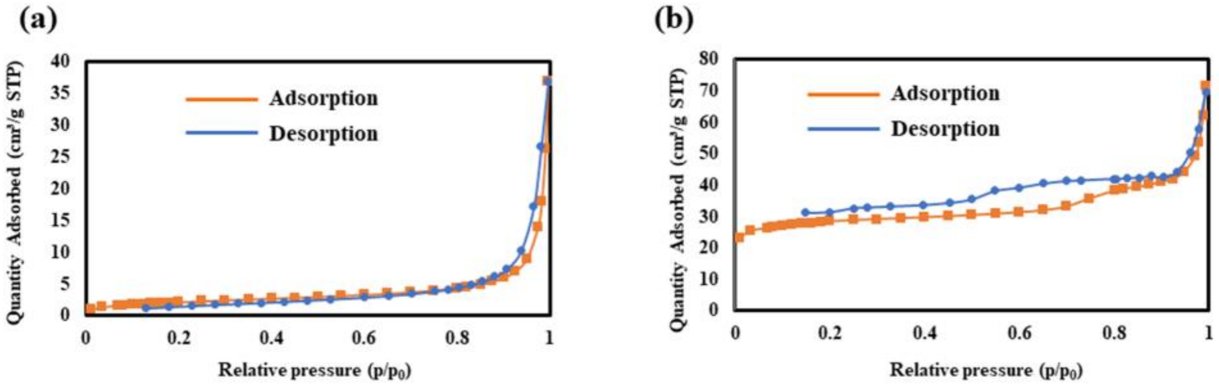

Fig. S7 *Nitrogen adsorption–desorption isotherms for* ***IUST-2****: (a) after the adsorption of Pb (II), (b) after removal Pb (II)*

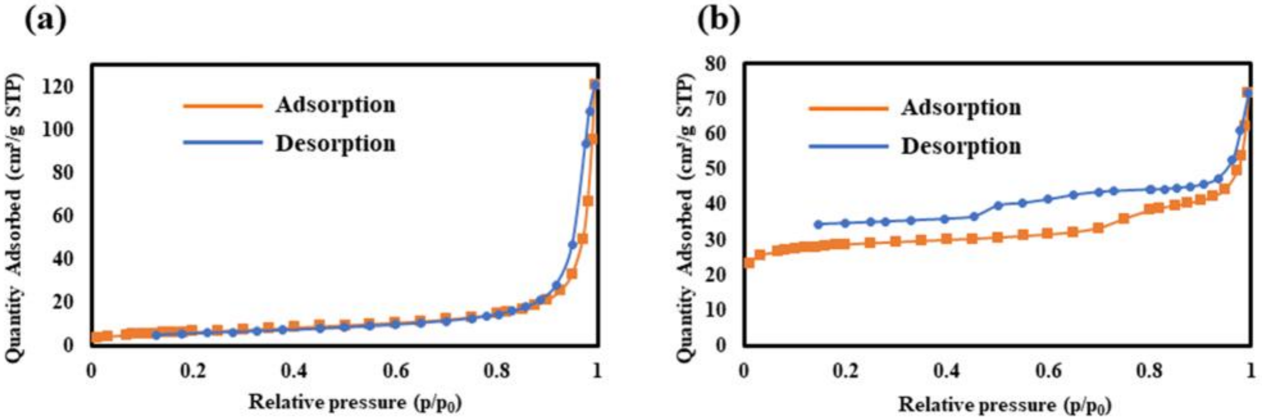

Fig. S8 *Nitrogen adsorption–desorption isotherms for* ***IUST-2****: (a) after the adsorption of Hg (II), (b) after removal Hg (II)*

Table S1 *Data Collection and Refinement Parameters for Single Crystal Analysis for* ***IUST-2***

Crystal data	
Chemical formula	$C_{42}H_{24}N_4O_{10}S_2Zn_2$
M_r	*939.51*
Crystal system, space group	*Orthorhombic, Pbcn*
Temperature (K)	*290*
a, b, c (Å)	*30.899 (6), 22.789 (5), 16.267 (3)*
α, β, γ (°)	*90, 90, 90*
V (Å^3)	*11455 (4)*
Z	*8*
Radiation type	*Mo Kα*
μ (mm^{-1})	*0.96*
Crystal size (mm)	*0.2 × 0.15 × 0.1*
Data collection	
Diffractometer	*MAR-345dtb*
No. of measured, independent and observed [I > 2σ(I)] reflections	*83563, 11729, 7099*
R_{int}	*0.079*
(sin θ/λ)$_{max}$ (Å^{-1})	*0.634*
Refinement	
$R[F^2 > 2\sigma(F^2)]$, $wR(F^2)$, S	*0.080, 0.251, 1.04*
No. of reflections	*11729*
No. of parameters	*541*
H-atom treatment	*H-atom parameters constrained* $w = 1/[\sigma^2(F_0^2) + (0.1152P)^2 + 15.3632P]$ $Whe\quad P = (F_0^2 + 2F_c^2)/3$
$\Delta\rho_{max}$, $\Delta\rho_{min}$ (e Å^{-3})	*0.87, −0.54*
CCDC number	*2181756*

Table S2 *Fitting results of Langmuir model for* ***IUST-2***

	q_m (mg·g^{-1})	b (L·mg^{-1})	R^2
Pb	1430	0.076	0.9933
Hg	900	0.049	0.9980

Table S3 *Fitting results of pseudo-second-order model for* ***IUST-2***

	Pseudo-second-order kinetic			
	$q_{e,\,exp}$ (mg·g^{-1})	k_1 (g (mg·min)$^{-1}$)	$q_{e,\,cal}$ (mg·g^{-1})	R^2
Pb	489.8	0.004	500	>0.9999
Hg	444.8	0.025	450	>0.9999

Table S4 *BET of* ***IUST-2*** *before and after removal of targeted metal ions*

BET plot	IUST-2	Pb		Hg	
		Before removal	After removal	Before removal	After removal
$a_{s,BET}$ [m^2 g^{-1}]	105.6356	6.3336	102.3546	18.2981	102.9832
Total pore volume(p/p_0=0.990) [cm^3 g^{-1}]	0.080687	0.020281	0.080431	0.065851	0.081760
Mean pore diameter [Å]	30.5528	128.0366	31.4321	143.9515	31.7566

Table S5 *Selected bond lengths (Å) of the* ***IUST-2***

Zn1—Zn2	2.8987 (8)	O2—C10	1.271 (6)
Zn1—O1^{i}	2.038 (4)	O6—C23	1.259 (7)
Zn1—O3	2.066 (4)	O8—C24	1.251 (6)
Zn1—O7	2.010 (4)	O5—C26	1.380 (7)
Zn1—O2^{ii}	2.049 (4)	O9—C38	1.381 (7)
Zn1—N1	2.030 (4)	N1—C7	1.341 (7)
Zn2—N4	2.015 (4)	N1—C17	1.318 (8)
S1—C5	1.711 (6)	C1—C10	1.485 (8)
S2—C41	1.727 (6)	N2—C5	1.345 (7)
O1—C12	1.260 (6)	C25—C39	1.353 (8)

Table S6 *Selected bond angles (^{0}) of the* ***IUST-2***

$O1^i$—Zn1—Zn2	80.86 (10)	N2—C5—S1	132.8 (6)
$O1^i$—Zn1—O3	89.76 (18)	C23—O6—Zn2	128.4 (4)
$O1^i$—Zn1—$O2^{ii}$	160.88 (15)	C7—N1—Zn1	123.5 (4)
N1—Zn1—Zn2	173.56 (14)	C17—N1—C7	117.9 (4)
N1—Zn1—$O1^i$	103.81 (17)	C32—O5—C26	119.0 (5)
N1—Zn1—O3	96.73 (18)	O4—C10—O2	124.8 (5)
O3—Zn1—Zn2	78.71 (11)	O4—C10—C1	117.3 (5)
O7—Zn1—Zn2	82.58 (11)	C34—C32—O5	117.2 (6)
